# Mental and behavioural disorders and COVID-19-associated death in older people

**DOI:** 10.1192/bjo.2020.87

**Published:** 2020-09-03

**Authors:** Billy Boland, Tim Gale

**Affiliations:** Hertfordshire Partnership University NHS Foundation Trust; and University of Hertfordshire, UK; Hertfordshire Partnership University NHS Foundation Trust; and University of Hertfordshire, UK

**Keywords:** Comorbidity, dementia, mortality, rating scales, COVID-19

## Abstract

Health factors such as diabetes, severe obesity and chronic kidney disease are all associated with a more severe outcome following coronavirus disease 2019 (COVID-19) infection. However, there has been little exploration into the impact of mental and behavioural disorders on outcomes associated with COVID-19. We investigated outcomes for older people who used mental health services. Those who had a COVID-19-associated death had previously rated worse across a range of health and social problems, including mental health related problems. Our findings evidence the need to urgently explore whether mental and behavioural disorders should also be considered a health risk factor for a more severe outcome from COVID-19 infection in older people.

## Background

The first case of coronavirus disease 2019 (COVID-19) in the UK was diagnosed at the end of January 2020. In the short time since, many thousands have died in the UK, with over 40 000 COVID-19-associated deaths reported at the beginning of June 2020.^1^ The UK government has defined groups of people who are considered ‘clinically vulnerable’. By this they mean people who are at ‘higher risk of severe illness from coronavirus’.^2^ People classified as clinically vulnerable include those who are over 70, those who are under 70 with one of a range of long-term physical health conditions such as diabetes, severe obesity or chronic kidney disease, and those who are pregnant.

People with mental and behavioural disorders of any severity are not currently considered clinically vulnerable. However, the life expectancy for people with severe mental illness continues to be worse than that of the general population, and the gap may be growing. Respiratory disorders account for some of this mortality.^3^

A recent Public Health England report into health inequalities associated with COVID-19 found that age brought the greatest disparity, with those aged over 80 years 70 times more likely to die than those under 40 years. Risk of dying was greater in males rather than females. Socially, deprivation was associated with a higher risk of COVID-19-associated death. Public Health England highlighted that dementia is included as a secondary diagnosis on a number of death certificates for those who had a COVID-19-associated death, but mental and behavioural disorders as a broad risk factor was not explored.^4^ There is an urgent need to better understand whether those who experience mental and behavioural disorders are at greater risk of dying from COVID-19.

## Method

The Health of the Nation Outcome Scale (HoNOS),^5^ is a clinician-rated outcome measure that is widely used in England. It is embedded within the National Health Service (NHS) mental health services data-set,^6^ and so its use in specialist mental health services is widespread. A 12-item scale, it was developed by the Royal College of Psychiatrists, and rates a range of health and social care factors, chiefly in secondary care. A variety of problems associated with mental and behavioural disorders are rated as separate items on the scale including problems associated with depressed mood, behavioural changes and hallucinations and/or delusions.

We have previously reported the development of a model for missing items on the HoNOS.^7^ We created separate models for adults of working age, and those over 65. Our model for the over 65s was based on HoNOS scores from 16 271 patients who used secondary mental healthcare services in in-patient and community settings in a single NHS trust.

We obtained the final HoNOS score for each of the people who had a COVID-19-associated death at this same NHS trust up to the 19 May 2020. HoNOS data was available for 58 individuals. The vast majority of these were over 65 (*n* = 52). There was complete HoNOS data (items 1–12) for 47 individuals, 4 had one missing item and 1 had two missing items. We estimated these six missing item values using our previously published model, rounding up or down to the nearest integer.

We compared this sample with our much larger reference sample of patients over 65 for whom HoNOS data was available, reported in our earlier paper.

## Results

The timing of the last HoNOS score for the cohort who died varied from 0 days (i.e. HoNOS was recorded on the day of death) to 1206 days. The median interval between date of last HoNOS and date of death was 111 days.

Our sample of people who experienced COVID-19-associated death included 35 men and 17 women, with a mean age of 81 years (s.d. = 7.6). The mean age of our larger reference sample was 82.7 years (s.d. = 6.5) and comprised 10 143 (62.3%) women and 6128 men (37.7%).

The COVID-19-associated death group had a higher proportion of men. The proportional difference is highly significant (χ^2^(d.f. = 1) = 18.18, *P* < 0.0001) and is line with other national and international findings that COVID-19-associated death is more likely in older men than older women. The COVID-19-associated death group had a similar mean age to the reference group.

Table 1 shows a comparison across HoNOS mean item scores and mean totals for the two groups. A one-sample *t*-test indicated that the COVID-19 group differed significantly from the reference group in terms of mean total HoNOS score (*t*(d.f. = 51) = 8.76, *P* < 0.0001). All item scores, except for item 3, differed significantly. However, controlling for multiple testing using Bonferroni correction (by dividing threshold *P* by number of individual comparisons), we would consider the prevailing significant differences to be on items 1, 4, 5, 6, 7, 8, 9, 10 and 12.

**Table 1:** Comparison of mean Health of the Nation Outcome Scale (HoNOS) item and total scores for COVID-19-associated death group and reference group^a^

HoNOS item	COVID-19-associated death group (*n* = 52)	Reference group^b^ (*n* = 16 271)	*P*
1. Overactive aggressive, disruptive or agitated behaviour	1.92	0.56	0.0001
2. Non-accidental self-injury	0.35	0.06	0.02
3. Problem drinking or drug taking	0.10	0.07	0.7 (NS)
4. Cognitive problems	2.83	2.11	0.0001
5. Physical illness or disability problems	2.23	1.65	0.0001
6. Problems with hallucinations and delusions	1.25	0.34	0.0001
7. Problems with depressed mood	1.31	0.75	0.001
8. Other mental and behavioural problems (specify)	1.96	0.85	0.0001
9. Problems with relationships	1.21	0.31	0.0001
10. Problems with activities of daily living	2.44	1.54	0.0001
11. Problems with living conditions	0.54	0.19	0.01
12. Problems with occupation and activities	1.29	0.68	0.002
Total HoNOS score	17.42	9.11	0.0001

NS, not significant.

a. HoNOS item scores for each item range from 0, no problem to 4, severe or very severe problem. The s.d. for the COVID and reference group total HoNOS score were 6.85 and 5.46 respectively. All *P-*values for item scores reflect one-sample *t*- tests comparing our COVID-19 group mean and s.d. with the reference group mean (d.f. = 51).

b. Our reference sample included two separate HoNOS ratings taken at different times for each patient.

## Discussion

### Main findings

It is clear that the COVID-19-associated death group have more highly rated health and social needs as compared with the reference group. This, however, is not consistent across all of the individual items. Some dimensions that were rated as greater problems in the COVID-19 group, such as physical health (item 5), and social problems (items 9, 10 and 12 (although note 11 was not significantly different)) have already been recognised by Public Health England as carrying greater risk for COVID-19-associated death. However, our data shows that the COVID-19-associated death group were also rated as having significantly worse mental health related issues such as problems with cognition, depressed mood, hallucinations and/or delusions, and behavioural problems.

An obvious explanation might be that the COVID-19-associated death group had a higher prevalence or greater severity of dementia, given this is a common diagnosis in this patient group. Nevertheless, those with other mental and behavioural disorders may also be at higher risk of infection from COVID-19, either as a direct result of their disorder, or the environment in which they receive care, such as a care home. Public Health England report that as of the 9 July 2020, there have been 6628 suspected or confirmed COVID-19 outbreaks reported in care homes.^8^

### Interpretation of our findings

The COVID-19 associated death group had their HoNOS score rated a median of 111 days prior to death. The maximum incubation period of COVID-19 has been estimated to be around 14 days,^9^ and the time from symptom development to death has been reported as 2–8 weeks.^10^ Therefore an upper limit from infection to death might be estimated to be 70 days (14 days + 8 weeks (56 days)). The median interval of 111 days between the final HoNOS rating and the date of death means, that for the majority of the COVID-19-associated death group, the last HoNOS score reflects their status prior to infection with COVID-19.

### Focus for future research

A recent position paper proposed that the research priorities for COVID-19 and mental health should focus on the impact of COVID-19 on mental health.^11^ However, our data suggests that in addition, the research community should also focus on whether people with pre-existing mental illness over the age of 65, such as people who use secondary-care mental health services, have a higher risk of severe illness from coronavirus:

- Are these people at greater risk of serious physical health consequences, including death, from COVID-19?
- How do those who have had a COVID-19-associated death differ from those who have recovered from COVID-19 in terms of mental and behavioural disorder problems?
- Can routine health outcome measures such as HoNOS identify people who are at higher risk of COVID-19-associated death among an already vulnerable population?

### Implications

We acknowledge the limitations and potential for confounding in our study, and recognise that our investigation raises more questions than answers. But these are important questions that require urgent exploration and are not currently getting sufficient attention. Any health inequalities that affect those who experience mental and behavioural disorders must be addressed as a priority.

## Data Availability

The data that support the findings of this study are available from B.B. upon reasonable request.
